# Formulation of Functional Drink with Milk Fortification: Effects on the Bioaccessibility and Intestinal Absorption of Phenolics

**DOI:** 10.3390/plants11233364

**Published:** 2022-12-03

**Authors:** Gulay Ozkan, Esra Capanoglu, Tuba Esatbeyoglu

**Affiliations:** 1 Department of Food Engineering, Faculty of Chemical and Metallurgical Engineering, Istanbul Technical University, Maslak, 34469 Istanbul, Türkiye; 2 Department of Food Development and Food Quality, Institute of Food Science and Human Nutrition, Gottfried Wilhelm Leibniz University Hannover, Am Kleinen Felde 30, 30167 Hannover, Germany

**Keywords:** rosehip, *Rosa canina*, catechin, matrix effect, in vitro digestion, bioaccessibility, Caco-2, absorption efficiency

## Abstract

Due to a turn toward to functional foods with improved nutritional value, rosehip could be an appropriate candidate to create formulations using a milk matrix. In the present study, the influence of bovine or almond milk fortification on the bioaccessibility and intestinal absorption of rosehip infusion phenolics, mainly catechin, were investigated by a combined method of an in vitro gastrointestinal digestion/Caco-2 cell culture model. The results indicated that bovine (IB) or almond milk (IA) fortification enhanced the retention of total phenolics (TPC; increase of 8.1% and 20.3% for IB and IA, respectively), while there was a decline in the total flavonoids (TFC; decrease of 64% and 17% for IB and IA, respectively) and antioxidant capacity measured by CUPRAC assay (decrease of 15% and 4% for IB and IA, respectively) throughout the gastrointestinal tract in comparison with the control sample (IC). Then, based on the cytotoxicity (SRB) assay, 1/5 times diluted digests were subjected to transepithelial transportation of Caco-2 cells. According to the results, the bovine milk matrix positively affected the transportation of phenolics across the epithelial cell layer. It could be concluded that it is possible to produce functional infusion drinks with improved stability, bioaccessibility, and absorption efficiency of rosehip phenolics in the formulations containing milk matrix.

## 1. Introduction

Foods which exhibit beneficial effects on one or more functions in the body by enhancing the health status and/or declining the risk status, beyond sufficient nutritive influences, can be described as “functional” [1]. There has been increasing attention paid to the design of functional foods by (i) fortification with ingredients that possess a beneficial effect, (ii) clarification of anti-nutrients, (iii) postharvest applications, or (iv) production of novel foods with an enhanced nutritional value [2]. However, there are some drawbacks that should be taken into consideration. It is not easy to incorporate nutraceuticals into food products due to their poor water solubility, crystalline structure, chemical instability during gastrointestinal tract, and low bioaccessibility and bioavailability [3,4]. Therefore, there have been some attempts to design the food matrix in order to reduce the factors limiting the bioavailability of bioactives [5]. For instance, particle-based delivery systems have been utilized in order to enhance the permeability and solubility of bioactive compounds [6]. Additionally, bioactive substances could be co-digested with an excipient food, or bioactive compounds may be added into the food product by mixing [5]. A recent study has reported that the stability and bioaccessibility of nutraceuticals obtained from tomato paste can be increased by co-ingestion with an excipient emulsion [7]. In this context, there has been some research investigating the bioaccessibility or bioavailability of bioactives in beverage systems fortified with bovine or plant-based milks, such as soy milk and almond milk, in order to improve the nutritional value of the product. For example, the metabolic fate of phenolics in cranberrybush; bovine or almond milk blends [8]; terebinth coffee-milk mixture [9]; apple, grape, or orange juice milk blends [10]; mixture of black tea and milk [11]; co-digested guaraná (*Paullinia cupana*) seed powder and casein [12]; as well as green tea catechin within a chocolate matrix [13], have been demonstrated previously.

The pseudo-fruits of *Rosa canina* (rosehip) have been recognized as a nutritional food due to their notable content of health-related bioactive compounds. Indeed, a variety of constituents have been reported for rosehip, such as vitamins, carotenoids, tocopherols, phenolic compounds, minerals, and essential oils [14,15]. Moreover, different kinds of food products could be produced using rosehip, including tea, infusion, juice, nectar, jam, marmalade, dried layers of fruit pulp, and vinegar [16,17].

There have been several investigations into the bioaccessibility of phenolic substances in milk blend formulations, but there is limited knowledge about the matrix effect on the bioavailability of phenolic compounds using the cell culture method. In the work explained here, we analyzed (i) the apparent phenolic concentrations in the infusion formulations prepared by bovine or almond milk; (ii) the bioaccessibility of infusion polyphenols after gastrointestinal digestion; (iii) the apparent antioxidant potential of the phenolics in infusion formulations; (iv) the change in the antioxidant properties of infusion phenolics during gastrointestinal digestion; (v) stability of catechin in the apical and basolateral sides of the cells; and (vi) intestinal absorption of catechin throughout the transepithelial transportation.

## 2. Results

### 2.1. Changes in the Phenolics and Their Antioxidant Potential during In Vitro Digestion

The influence of the milk matrix and simulated in vitro digestion on the retention of rosehip bioactives are presented in Table 1. Considering the fortification of rosehip infusion with bovine or almond milk matrix, before digestion, significant increases were determined in the apparent TPC and TFC amounts in IB, as well as TFC in IB and IA, in comparison to that of the IC sample (*p* < 0.05).

With regard to the effect of digestion simulation, the retention rates of TPC and TFC were recorded as 74.9 and 57.3%, respectively, in the IC sample. In contrast, the almond milk fortification significantly enhanced the recovery of TPC (88.6%) in comparison to the IC and IB samples (*p* < 0.05). In addition to these, there was a remarkable decrease in the bioaccessibility of TFC by addition of bovine (20.1%) or almond milk (38.5%) when compared to the IC sample (57.3%) (*p* < 0.05).

Regarding the catechin content, changes in the apparent concentration and retention after digestion are shown in Table 2. Before digestion, a significant decrease was observed in the apparent concentration in the IB (3551 mg/100 g) and IA (3307 mg/100 g) samples in comparison to that of the IC sample (4159 mg/100 g) (*p* < 0.05). Similarly, the bioaccessibility of catechin content was found to be higher in the IC sample (81.3%), while there was an effective decrease in the retention of catechin in the IB (67.9%) and IA (71.4%) samples (*p* < 0.05).

In order to reveal the effects of the milk matrix and simulated in vitro digestion on the antioxidant potential of rosehip infusion phenolics, data obtained from DPPH and CUPRAC assays are depicted in Table 3. Based on apparent values, while the fortification of rosehip infusion with the milk matrix (IB and IA) did not alter the DPPH radical scavenging activity (*p* > 0.05), CUPRAC values were significantly ascended in the IB (8756 mg TE/100 g) and IA (7407 mg TE/100 g) samples, but not in the IC (6521 mg TE/ 100 g) infusion sample (*p* < 0.05).

After in vitro gastrointestinal digestion, the increases in the DPPH radical scavenging activity of IC, IB, and IA infusions were found to be 2.5, 2.8, and 3.1-fold, respectively. It is clear that the addition of a milk matrix (IB or IA) improved the recovery of DPPH antioxidant capacity. In contrast, the bioaccessibility of CUPRAC antioxidant capacity values was not significantly affected by the milk matrix (*p* > 0.05).

### 2.2. Caco-2 Cytotoxicity

**Figure 1:**
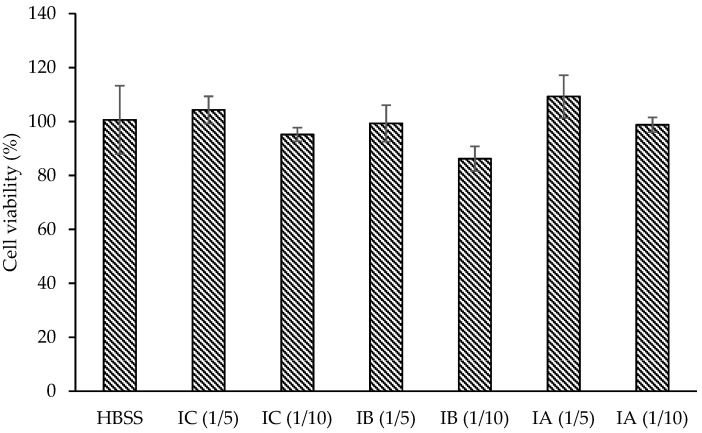
Cell viability (%) of treated samples. IC: infusion control sample, IB: infusion with bovine milk blend, IA: infusion with almond milk blend.

Before transepithelial transportation, the maximum non-toxic sample amount should be determined. Therefore, the viability of sample treated cells was obtained by means of SRB assay, in which cellular protein content was analyzed (Figure 1). The cell viability was always found to be greater than 85%, which means there was no major cytotoxic effect of 1/5 and 1/10 times diluted digests (*p* > 0.05). Thereby, the dilution ratio was fixed to 1/5 in further transport experiments.

### 2.3. Transport Experiments

Control of the Caco-2 cell monolayer integrity was performed by measuring the TEER values of the cells during a 21-day differentiation period, before the experiment as well as at the end of 4 h incubation and after 24 h of the transportation. Results of the TEER values of Caco-2 cells are presented in Table 4. TEER values higher than 300 Ωcm^2^ (average initial value) were selected for the transport experiments. According to the results, 1/5 times diluted digests in HBSS showed higher retained values, described as a ratio between the TEER value of the cells after incubation and the TEER value of the cells before incubation.

**Figure 2:**
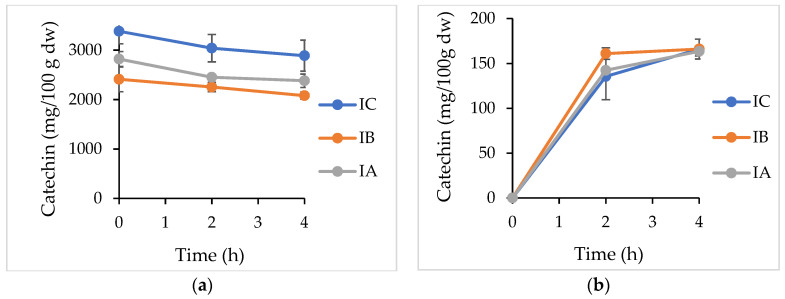
The apical (**a**) and basolateral (**b**) side concentration of catechin during 4 h of transportation. IC: infusion control sample, IB: infusion with bovine milk blend, IA: infusion with almond milk blend.

The metabolic fate of catechin in the infusions was determined by sampling from both apical and basolateral sides of the cells during a 4-hour transepithelial transportation assay. The amount of catechin in the apical and basolateral sides during incubation, as well as its absorption efficiency after incubation, are reported in Table 2 and Figure 2. Results indicated that the amount of catechin in the apical compartment after 2 h of treatment was obtained as 3041, 2254, and 2254 mg/100 g, whereas it was decreased to 2889, 2080, and 2382 mg/100 g for IC, IB, and IA, respectively. Furthermore, the stability of catechin in IC was statistically higher than at in IB and IA (*p* < 0.05) during the 4-hour exposure time. On the other hand, the amount of catechin in the basolateral side after 2 h of transportation was calculated as 135, 161, and 142 mg/100 g, while it increased to 166, 166, and 163 mg/100 g for IC, IB, and IA, respectively. Moreover, the difference in catechin content of infusions at 2 or 4 h was not found to be statistically significant (*p* > 0.05). In addition, outcomes regarding the catechin absorption efficiency, or the transportation of catechin from the apical (t = 0 h) to the basolateral side (t = 4 h), demonstrated a higher bioavailability of catechin in IB infusion compared to the IC and IA samples (Table 2) (*p* < 0.05).

## 3. Discussion

For undigested samples, the spectrophotometric quantification of the total phenolics and flavonoids in infusion samples showed that the fortification with bovine milk matrix enhanced the extractability of rosehip phenolics and flavonoids significantly (*p* < 0.05), whereas fortification with almond milk only enhanced the apparent amounts of flavonoids (*p* < 0.05). Likewise, according to Kamiloglu et al. [9], total phenolics in terebinth coffee and whole or skim milk matrix blends were found be higher (131–160%) than those of the control sample, regardless the type of milk matrix. In contrast, the trends obtained for undigested infusion formulations were not in line with the results observed during digestion. At each step of the digestion, there was a decrease in the results, due to the polymerization, oxidative degradation, transformation, and complexation with proteins and metal ions during digestion [10]. Moreover, the highest bioaccesibility of TPC was obtained with the IA infusion, whereas the bioaccessibility of TFC was decreased significantly with the addition of the milk matrix. This remarkable reduction may be due to the complexation between the procyanidins in rosehip infusions and the proline-rich casein protein in milk at the pH value around the isoelectric point of the protein [18]. On the other hand, there was an interesting difference between the results of the present study and the findings from the cranberrybush juice and milk blends, which were obtained previously by our research team. While the bovine milk matrix caused a decrease in rosehip bioactives during digestion, the interaction between cranberrybush phenolics and milk fat may have contributed the transfer of phenolics into micelles. In addition to this, it was indicated that some of the bound cranberrybush phenolics, because of the interaction between cranberrybush phenolics and milk protein, could be recovered during digestion by the action of digestive enzymes [8].

Although chlorogenic acid, catechin, quercetin, and rutin have been determined as the major phenolic substances in rosehip [19,20], only catechin was detected after simulated in vitro gastrointestinal digestion [19]. Therefore, in this study, in order to monitor the effects of the milk matrix and digestion on the individual phenolic compounds, catechin content was analyzed. Results obtained from the chromatographic quantification of catechin were in line with the those from the TFC of samples; the bioaccessibility of catechin was found to be lower in the IB and IA samples in comparison with the IC sample. Furthermore, it is possible to observe the effect of milk protein–rosehip phenolics interactions in the apparent value of catechin in undigested samples, as well. It is likely that there is a decrease in the bioaccessibility of chlorogenic acid in cranberrybush, bovine, or almond milk blends [8]; chlorogenic acid; *p-*coumaric acid; quercetin; hesperidin; naringenin; catechin; and rutin in orange, kiwi, pineapple, and mango juice mix blended with bovine milk [21]. 

According to the results of the DPPH radical scavenging activity assay, there was no significant difference in the apparent values before digestion. However, this value was calculated to be much higher (2.5–3.1-fold) after gastrointestinal digestion compared to undigested samples, indicating an improvement in the DPPH radical scavenging capacity of the phenolic compounds due to the deprotonation of the hydroxyl groups in the aromatic rings at higher pH values [22,23]. Similar results were also reported previously for the quercetin and rutin microparticles [24], for some herb polyphenols [25], and for grape polyphenols [26]. On the other hand, the data obtained from the CUPRAC assay were found be compatible with the results of the flavonoid content of the undigested samples. The CUPRAC antioxidant potential of the infusions was enhanced with the addition of a milk matrix before digestion. In contrast, after gastrointestinal digestion, even though there was a significant drop in the content of flavonoids, an increasing tendency (not significant) was observed in the CUPRAC values. This phenomenon may be related to the formation of tyrosine and tryptophan as free amino acids as well as peptides, which possess antioxidant capacity by the proteolysis of milk proteins [27]. In the literature, ascended antioxidant potential after digestion has been indicated for cinnamon-milk [26], kiwi, pomelo juice-milk/soymilk [28], apple, grape, or orange juice blends with milk [10].

Due to the high connection between the in vivo and in vitro approaches to investigating the metabolic fate of plant phenolics [29], the Caco-2 human colon adenocarcinoma cell line widely takes part in the prediction of bioavailability studies [30,31]. Before transepithelial transportation assay, the non-toxic maximum sample amount and TEER values of the cells should be controlled in order to protect the cell integrity during the treatment of cells with samples. In this study, 1/5 of dilution ratio was selected in transport studies due to the absence of a loss of cells from the monolayer. As for the transportation of catechin in the infusions, while the stability of catechin on the apical side was decreased significantly after 4 h of incubation in all formulations, the IC sample always contained a higher amount of catechin (*p* < 0.05) in comparison to that of the IB and IA samples. Moreover, at the end of incubation, the catechin concentration reached 166, 166, and 163 mg/100 g in IC, IB, and IA, respectively. In more detail, the higher absorption efficiency of catechin in IB (6.89%) is likely linked to a difference in the protein and fat content of the matrices. The outcomes of the present research are in agreement with those obtained previously. The transport efficiency of chlorogenic acid as a primary phenolic compound in cranberrybush juice was affected in a positive manner using bovine or almond milk matrices [8]. Moreover, in another examination, the absorption of chlorogenic acid in coffee was recorded as higher (0.25%), but not significant, in a semi-skimmed milk blend rather than the control group without a milk matrix (0.14%) [32]. In addition to these studies, guaraná (*Paullinia cupana*) seed powder, which is rich in catechin, epicatechin, procyanidin B1, and procyanidin B2 was used to evaluate the cell permeability of those phenolic compounds as a response to co-digestion with macronutrients, such as casein, potato starch, and vegetable oil. The results showed that there was no impact on the bioavailability of catechins in the presence of casein [12]. However, one study claimed that milk could diminish the bioavailability of black tea catechins [11]. The other study also reported a reduced absorption of green tea catechins by the chocolate matrix, indicating an inhibitory effect of protein (7.6% in chocolate) and fat (33.8% in chocolate) as a chocolate constituent [13].

In addition to this, regarding the effects of catechins on the tight junction barrier properties, it has been reported that the increase in the permeability of Caco-2 cells was in line with the disruption of barrier properties of tight junctions by the treatment of samples containing catechin or catechin derivatives. Moreover, there was a tendency of the paracellular transport to increase with the application of some food samples, even without any evidence of cytotoxicity [33].

It could be deduced from the findings gained here that it is possible to produce functional drinks with improved stability and intestinal absorption of phenolic compounds by way of using milk fortification. For an exhaustive inference, it could be suggested to evaluate the sulfated, glucuronidated, or methylated metabolites of catechin formed during each stage of the digestion and transportation by varying the ratio between infusion and milk.

## 4. Materials and Methods

### 4.1. Materials and Chemicals

Dried rosehip samples were obtained from a local supplier in Bursa, Türkiye. 

All chemicals were of analytical or HPLC-grade. Enzymes of pepsin from porcine gastric mucosa and pancreatin from porcine pancreas, as well as bile for the simulated in vitro gastrointestinal digestion, were provided from Sigma–Aldrich (Schnelldorf, Germany). Ultrapure (Purelab flex 3; VeoliaWater Technologies, Celle, Germany) or MQ water was used for the analyses.

### 4.2. Sample Preparation

Infusions from dried and ground rosehip were prepared according to Ozkan et al. [19]. The milk products, including bovine or almond, were supplied from a local market in Hannover, Germany. The nutritional composition of bovine whole milk consists of 3.6% (*w/v*) fat, 3.3% (*w/v*) protein, and 4.8% (*w/v*) carbohydrates. Almond milk contains 1.1% (*w/v*) fat, 0.5% (*w/v*) protein, and 0.2% (*w/v*) fibre (data provided by manufacturers). In order to investigate impact of the milk matrix on the phenolic bioaccessibility of the infusions, three different formulations were prepared by mixing 75% infusion and 25% water (IC; infusion control), bovine milk (IB; infusion with bovine milk blend), or almond milk (IA; infusion with almond milk blend). All formulations were prepared in triplicate and immediately subjected to gastrointestinal digestion simulation.

### 4.3. In Vitro Gastrointestinal Digestion

A simulation of the physiological conditions of in vitro digestion in the human stomach and intestine was conducted based on Sessa et al. [34] and Ozkan et al. [8]. Each mixture, containing five milliliters of sample and 5 mL of phosphate buffer saline, was preincubated in a water bath at 37 °C and 100 rpm for 15 min. 

For the simulation of gastric digestion, the pH value of the infusions was fixed to 2.0 by adding 1 M HCl and pepsin solution at 1.3 mg/mL. The final concentrations were added to the samples. Afterwards, mixtures were placed into a water bath at 37 °C and 100 rpm for 2 h.

For the simulation of intestinal digestion, the pH of the remaining fluid was adjusted to 5.8 by adding 1 M NaHCO_3_; then, 2.5 mL of pancreatin and bile salts solution mixture, at 0.175 and 1.1 mg/mL final concentrations, respectively, were added to the mixture. Samples were placed into the water bath at 37 °C and 100 rpm for 2 h after increasing the pH to 5.8 by adding 1 M NaHCO_3_.

A blank without the infusion formulations was also subjected to in vitro gastrointestinal digestion under the same physiological conditions, in order to eliminate any interferences from the digestive fluids.

After simulation of digestion, the samples were cooled forthwith by dipping into an ice bath. A residual fraction of the digests was separated from the bioaccessible fraction by centrifugation (Megafuge 8R; Thermo Scientific, Darmstadt, Germany) at 10,000 rpm and 4 °C for 30 min. Bioaccessible fractions were stored at −80 °C until spectrophotometric, chromatographic, and Caco-2 cell culture experiments. All experiments were performed in triplicates. 

The bioaccessibility of phenolics was determined by dividing the concentration of phenolics in the bioaccessible fraction to the concentration of phenolics in the initial infusion formulation.

### 4.4. Spectrophotometric Analysis

Total phenolic content (TPC), total flavonoid content (TFC), and total antioxidant capacity (TAC) of the samples were detected using an UV–Vis spectrophotometer (Infinite M200, Tecan, Crailsheim, Germany).

The TPC assay was performed using the Folin–Ciocalteu reagent [35]. The outcomes were reported as mg gallic acid equivalents (GAE) per 100 g dry weight (dw) (R^2^ = 0.9986, y = 4.5642x + 0.0262). 

The TFC assay was performed according to Dewanto et al. [36]. The results were stated as mg rutin equivalents (RE) per 100 g dw (R^2^ = 0.9913, y = 1.9817x − 0.0674).

The TAC of the samples was determined by CUPRAC [37] and DPPH [38] assays. The results were given as mg Trolox equivalents (TE) per 100 g dw (R^2^ = 0.9984, y = 1.0097x + 0.0019 for CUPRAC and R^2^ = 0.9916, y = 0.0086x − 0.0277 for DPPH).

### 4.5. Cell Culture

The human colon adenocarcinoma Caco-2 cells (German Collection of Microorganisms and Cell Cultures, Braunschweig, Germany) were cultured in Dulbecco’s modified Eagle’s medium (DMEM), containing 4.5 g/L glucose and stable glutamine, supplemented with 20% fetal bovine serum (FBS), 1% non-essential amino acids (100x), 100 IU/mL penicillin, and 100 µg/mL streptomycin (Pan Biotech, Aidenbach, Germany) at 37 °C in a 5% CO_2_ humidified atmosphere. Cultured cells were split once they reached confluence using trypsinization (trypsin/EDTA (10x), Pan Biotech, Aidenbach, Germany).

### 4.6. Cytotoxicity Assay

Caco-2 cells were seeded in 96-well plates and left for 14 days after confluency. Then, cells were treated with digests, obtained after simulated gastrointestinal digestion, at a dilution ratio of 1/5 and 1/10 (*v/v*) in HBSS. After standing for 4 h, cytotoxicity was determined by the sulforhodamine B (SRB) assay [39]. 

### 4.7. Transport Experiment

For the transport experiment, Caco-2 cells were seeded at a density of 4 × 10^4^ cells/well in 6-well transwell plates inserts (0.4 µm pore diameter, 24 mm insert, Sarstedt, Nümbrecht, Germany). Then, 2 mL and 2.5 mL of growth medium were added to the apical and basal compartments, respectively. Before the transepithelial transport studies, cells were cultured for 21 days to allow for differentiation. The integrity of the cell layers was assessed by monitoring the transepithelial electrical resistance and TEER value (Millicell ERS-2 Volt-Ohm Meter, Millipore, Bedford, MA, USA). Subcultured cells with TEER values that were consistent and greater than 200 Ωcm^2^ [40] were utilized in the transport studies. The Caco-2 cells treated in the current study possessed passage numbers of less than 30.

The transportation efficiency of phenolics in formulated infusion samples was evaluated according to Ozkan et al. [30] and Wu et al. [31]. After differentiation, the transport experiment began with the replacement of the growth medium in the cell compartments with HBSS. After incubation with HBSS for 1 h, HBSS was replaced with a bioaccessible fraction of digests at 1/5 dilution ratio (results obtained from SRB assay; Section 2.2). Transport experiments were carried out at 37 °C with 5% CO_2_ in air for 4 h. Throughout the experiment, 200 µL of samples were collected from the upper and lower compartments of the transwell at 2 h intervals, and then filled with 200 µL of HBSS after sample collection. In order to control the monolayer cell integrity, TEER values were monitored before and after the experiment. At the end of transportation, digests were replaced with growth medium and left to incubate for another 20 h to determine any irreversible injury in the integrity of the cell layer. All experiments were performed in triplicates and samples were stored at −80 °C until further analysis.

The absorption efficiency of catechin was determined by dividing the amount of catechin in the basolateral side after 4 h by the amount of catechin in the apical side at 0 h.

### 4.8. Quantification of catechin by UPLC-UV

Catechin was quantified based on a procedure reported previously by Ozkan et al. [15]. Prior to the injection to the UPLC system (Waters Co., Milford, MA, USA), samples were filtered through a 0.20 µm membrane. The UPLC system includes a UPLC BEH Phenyl column 2.1 × 100 mm, 1.7 µm (Waters Acquity, Milford, MA, USA) as stationary phase and tunable UV detector. The mobile phase consists of formic acid in MQ water (1/1000, *v/v*; eluent A) and formic acid in methanol (1/1000, *v/v*; eluent B). The linear gradient was conducted as follows: 0 min, 10% B; 0–6.48 min, 65% B; 6.48–6.77 min, 100% B; 6.77–8.60 min, 100% B; and 8.60–8.70 min, 10% B at 0.6 mL/min of flow rate and 10 μL of injection volume). Spectral measurements were performed at 280 and 330 nm. Catechin was calculated using its authentic standard. A calibration curve, in the range of 0.012–0.125 mg/mL, was plotted according to nominal concentrations versus chromatographic peak area (y = 66708x). All results were stated as mg catechin per 100 g dw.

### 4.9. Statistical Analysis

All experiments were performed at least in three replicates. Results were given as mean ± standard deviation. Statistical analysis was conducted using SPSS software (version 20.0, SPSS Inc. Chicago, IL, USA). Formulations were evaluated by using one-way analysis of variance (ANOVA) followed by a Tukey post hoc test (*p* < 0.05).

## Figures and Tables

**Table 1:** Retention of phenolics in the formulations throughout in vitro digestion.

Sample	TPC (mg GAE/100 g)			TFC (mg RE/100 g)		
	Undigested	Digested	Bioaccessibility (%)	Undigested	Digested	Bioaccessibility (%)
IC	2555 ± 77 ^b^	2097 ± 222 ^b^	74.9 ± 4.50 ^b^	1603 ± 39 ^c^	964 ± 70 ^a^	57.3 ± 1.00 ^a^
IB	3266 ± 104 ^a^	2494 ± 275 ^a^	78.8 ± 6.00 ^b^	3911 ± 90 ^a^	788 ± 51 ^b^	20.1 ± 1.30 ^c^
IA	2522 ± 72 ^b^	2302 ± 96 ^ab^	88.6 ± 1.50 ^a^	2484 ± 68 ^b^	957 ± 122 ^a^	38.5 ± 4.90 ^b^

Small letters in the columns show statistically considerable variations within the formulations (*p* < 0.05). GAE: gallic acid equivalents, RE: rutin equivalents, IC: infusion control sample, IB: infusion with bovine milk blend, IA: infusion with almond milk blend.

**Table 2:** Changes in catechin content of rosehip infusions.

Sample	Undigested (mg/100 g dw)	Bioaccessibility (%)	Absorption Efficiency (%)
IC	4159 ± 400 ^a^	81.3 ± 0.60 ^a^	5.09 ± 0.53 ^b^
IB	3551 ± 306 ^ab^	67.9 ± 0.50 ^b^	6.89 ± 0.11 ^a^
IA	3307 ± 11 ^b^	71.4 ± 2.30 ^b^	5.79 ± 1.02 ^ab^

Small letters in the columns show statistically considerable variations within the formulations (*p* < 0.05). IC: infusion control sample, IB: infusion with bovine milk blend, IA: infusion with almond milk blend.

**Table 3:** Changes in the antioxidant potential of infusion phenolics throughout in vitro gastrointestinal digestion.

Sample	DPPH (mmol TE/100 g dw)			CUPRAC (mg TE/100 g dw)		
	Undigested	Digested	Increase (Fold)	Undigested	Digested	Bioaccessibility (%)
IC	6183 ± 658 ^a^	16192 ± 494 ^b^	2.5	6521 ± 455 ^c^	5160 ± 563 ^a^	70.9 ± 8.9 ^a^
IB	6229 ± 121 ^a^	17577 ± 1327 ^ab^	2.8	8756 ± 263 ^a^	5092 ± 438 ^a^	72.5 ± 6.4 ^a^
IA	6028 ± 211 ^a^	18198 ± 1575 ^a^	3.1	7407 ± 237 ^b^	5562 ± 476 ^a^	75.1 ± 6.4 ^a^

Small letters in the columns show statistically considerable variations within the formulations (*p* < 0.05). IC: infusion control sample, IB: infusion with bovine milk blend, IA: infusion with almond milk blend.

**Table 4:** TEER (Ωcm^2^) values of Caco-2 cells were measured at 0 h and after 4 h and 24 h of incubation.

Sample	After 4 h of Transportation	After 24 h of Transportation
HBSS (without digest)	300 ± 0.00	350 ± 7.07
IC	300 ± 0.00	300 ± 0.00
IB	310 ± 17.32	333 ± 28.87
IA	300 ± 0.00	306 ± 11.55

IC: infusion control sample, IB: infusion with bovine milk blend, IA: infusion with almond milk blend.

## Data Availability

Not applicable.
